# Molecular Topology for the Search of New Anti-MRSA Compounds

**DOI:** 10.3390/ijms22115823

**Published:** 2021-05-29

**Authors:** Jose I. Bueso-Bordils, Pedro A. Alemán-López, Rafael Martín-Algarra, Maria J. Duart, Antonio Falcó, Gerardo M. Antón-Fos

**Affiliations:** 1 Departamento de Farmacia, Universidad Cardenal Herrera-CEU, CEU Universities C/Ramón y Cajal s/n, 46115 Alfara del Patriarca, Valencia, Spain; paleman@uchceu.es (P.A.A.-L.); rmartin@uchceu.es (R.M.-A.); mduart@uchceu.es (M.J.D.); ganton@uchceu.es (G.M.A.-F.); 2 ESI International Chair@CEU-UCH, Departamento de Matemáticas, Física y Ciencias Tecnológicas, Universidad Cardenal Herrera-CEU, CEU Universities San Bartolomé 55, 46115 Alfara del Patriarca, Valencia, Spain; afalco@uchceu.es

**Keywords:** antibacterial, antibiotics, anti-MRSA, computational chemistry, linear discriminant analysis, molecular topology, molecular connectivity, topological indices, quinolones, QSAR

## Abstract

The variability of methicillin-resistant *Staphylococcus aureus* (MRSA), its rapid adaptive response against environmental changes, and its continued acquisition of antibiotic resistance determinants have made it commonplace in hospitals, where it causes the problem of multidrug resistance. In this study, we used molecular topology to develop several discriminant equations capable of classifying compounds according to their anti-MRSA activity. Topological indices were used as structural descriptors and their relationship with anti-MRSA activity was determined by applying linear discriminant analysis (LDA) on a group of quinolones and quinolone-like compounds. Four extra equations were constructed, named DF_MRSA1_, DF_MRSA2_, DF_MRSA3_ and DF_MRSA4_ (DF_MRSA_ was built in a previous study), all with good statistical parameters, such as Fisher–Snedecor F (>68 in all cases), Wilk’s lambda (<0.13 in all cases), and percentage of correct classification (>94% in all cases), which allows a reliable extrapolation prediction of antibacterial activity in any organic compound. The results obtained clearly reveal the high efficiency of combining molecular topology with LDA for the prediction of anti-MRSA activity.

## 1. Introduction

*Staphylococcus aureus* is a Gram-positive bacterium that causes important infections in humans. The first strain of methicillin-resistant *Staphylococcus aureus* (MRSA) was identified in 1960, almost immediately after the introduction of methicillin in therapeutics [1,2].

Antibiotic resistance occurs when the bacteria that cause the infection survive after exposure to a drug that, under normal conditions, would kill or inhibit its growth [3]. As a result, these surviving strains multiply and spread, due to the lack of competition from other strains sensitive to the same drug. This has led to the emergence of what we call “superbugs”, such as MRSA, which are difficult to treat with available antibiotics [4].

MRSA is an important cause of infections, both of community and hospital origin, and represents a major clinical and public health problem due to the limited treatment options (partly due to its worldwide spread), well-established resistance to vancomycin (formerly the antibiotic of choice for MRSA infections), and high number of therapeutic failures [5,6]. In fact, the World Health Organization (WHO) has placed MRSA as a priority 2 (high-priority) pathogen on its list of priority pathogens for the development of new antibiotics [7]. Furthermore, according to the latest antibacterial resistance report developed by the European Center for Disease Prevention and Control (ECDC), MRSA strains are present in virtually the entire European continent, being one of the most common causes of nosocomial infections [8].

There are several factors that contribute to the success of this bacterium as a pathogen, among which are its ability to persist as a commensal, its frequent resistance to multiple antibacterials, and its variety of virulence determinants [9]. These bacteria have a great capacity to survive in adverse environments, which, after entering the hospital environment through patients, visitors, and/or healthcare workers, spread to other patients, mainly through the hands of healthcare personnel [10]. However, although initially related to the hospital setting, MRSA infections are common at the community level as well [11].

Its morbidity is variable and depends on factors specific to the host, the type of infection, and the precociousness of treatment. Although most of these infections affect the skin and soft tissues, these organisms are also capable of causing devastating diseases in certain patients. These infections include necrotizing fasciitis, septic thrombophlebitis of the extremities, Waterhouse–Frederickson syndrome, and rapidly progressive pneumonia [12].

Hence, MRSA can be regarded as a serious health problem worldwide. The current interest in the study of this pathogen is due to its high frequency and because it represents one of the main causes of nosocomial infection outbreaks around the world. The growing increase in resistant strains, which is now spreading faster in comparison to the development of new molecules, makes it necessary to investigate new antibacterial agents to expand the current therapeutic arsenal. In addition, WHO has repeatedly noted that current investment in the development of new antimicrobial compounds is insufficient [13].

Empirically establishing rules or filters for drugs, such as Lipinski’s rule of five, will form the knowledge base to produce libraries tailored to drug discovery, which will require high-throughput identification of novel compounds within a large background of known substances [14].

This could be achieved by means of a rational drug design approach such as Quantitative Structure–Activity Relationships (QSAR). Within this field is molecular connectivity or molecular topology (MT), an effective and low-cost method capable of predicting molecular properties in new compounds, without the need to obtain or synthesize them previously [15].

By combining MT with pattern recognition techniques such as linear discriminant analysis (LDA) [16], neural networks [17], multilinear regression [18], factor analysis [19], or principal component analysis [20], and appropriately selecting the molecular descriptors to use, we can build mathematical–topological equations able to identify almost any molecular property. This makes MT a powerful tool for the search and design of new compounds with antibacterial activities, but it is not the only one in the field. In fact, a variety of linear and nonlinear statistical methods are used to develop models based on 2D or 3D representations of molecules [21]; 1D–3D-QSAR methods pose a series of limitations that have led to the development of higher dimensional QSAR models (4D–7D). Multi-dimensional models, although technically more complex, have been developed with the objective of finding the true binding mode [22].

In this context, this study aims to obtain mathematical–topological equations capable of predicting anti-MRSA activity. By combining MT and LDA, we use topological indices (TI) [23] to classify a compound as anti-MRSA or non-anti-MRSA. To do this, we simply select a group of compounds with antibacterial activity and another one lacking it.

**Figure 1:**
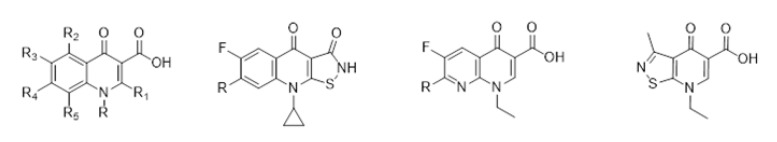
Core structures of the compounds used for the realization of the equations.

We have selected structures from quinolones and quinolone-like compounds to build these equations (see Figure 1). Quinolones are a well-known and extensive group that will allow us to collect numerous data, leading to greater precision of the predictive equations [24]. In addition, their pharmacokinetic profile, as well as their high antibacterial activity and broad spectrum of action, makes them very versatile [25]. Furthermore, compared with the resistance levels of other classes of antibacterial, those of quinolones are relatively low [26].

The discriminant functions obtained can be applied to quinolones and quinolone-like compounds not used in the study as well as in molecules that have no structural relationship, since they will select those that have a similar mathematical–topological relationship [27]. Since topology is the part of the mathematical analysis that studies the positions and interconnections of elements within a set, when applied to molecules, it gives rise to the discipline called MT, which analyzes the positions and interconnections of atoms within a molecule, giving structural information regarding length, ramifications, connection between atoms, shape, instaurations, etc.—in short, to the topological assembly or connectivity of the molecule [28]. Structurally related compounds will usually have similar values for their topological indices, but this can also occur in compounds with non-related structures.

The effectiveness of the topological method has also been proven, having been successfully applied to different therapeutic groups, such as anti-HIV [29], antihistaminic [30], antitumor [31], anti-Alzheimer [32], and antibacterial agents [33].

## 2. Results

To obtain the discriminant functions (DF), we used the data from a previous study [34], in which the statistical program BMDP randomly formed two training groups with 26 active and 30 inactive compounds and two test groups with seven active and six inactive compounds. These test groups allowed evaluation of the quality of the selected functions.

The DFs formed in this study along with their statistical parameters are shown in Equations (1)–(4), while their corresponding classification matrices are shown in Table 1, Table 2, Table 3 and Table 4:

DF_MRSA1_ = 18.11717 − 3.16799*S-_CH3_* + 8.24096*S_=C<_* − 6.48843*S_aSa_* + 1.91821*S_Cl_* N = 43 λ = 0.1223510 F = 68.145(1)

DF_MRSA2_ = 62.90981 − 15.34013*S_>CH-_* + 14.60322*S_=C<_* + 3.06032*S_Cl_ −* 220.70956^3^*J^V^* N = 43 λ = 0.0865409 F = 100.275(2)

DF_MRSA3_ = 45.25057 − 3.70040*S_>N-_* + 3.76891*S_Cl_* − 400.80700^3^*J* + 3.60644*PR2* N = 43 λ = 0.0894309 F = 96.727(3)

DF_MRSA4_ = 70.32771 + 84.92148^3^*χ_ch_* − 16.26568*S_-CH=_* + 3.69496*S_Cl_ −* 394.47199^3^*J* N = 43 λ = 0.0628582 F = 141.634(4)

The classification criterion was determined by the value of the DF: if the value of the equation for a given compound was equal to or greater than 0, such a compound was classified as active, whereas if the value of the equation for a compound was smaller than 0, such a compound was classified as inactive.

## 3. Discussion

All equations have a low value of λ, indicating that there is a low linear dependence between independent variables. Furthermore, the high value of F in the equations indicates that the selected independent variables contribute largely to the separation of the active and inactive groups. Moreover, all equations correctly classify each compound with its corresponding group with very high success rates (100% in most cases).

We obtained the first three functions using combinations of different types of indices: DF_MRSA1_ used the electrotopological state (*S_i_*) [35]; DF_MRSA2_ used electrotopological and charge indices [36]; while DF_MRSA3_ used all topological indices excluding the connectivity indices [37]. We obtained DF_MRSA4_ using all 136 topological indices.

DF_MRSA1_ involves four electrotopological indices (*S-_CH3_*, *S_=C<_*, *S_aSa_* and *S_Cl_*). Its value is influenced positively by the presence of sp^2^ carbons (*S_=C<_*) and chlorine atoms (*S_Cl_*). However, the presence of methyl groups (*S-_CH3_*) and sulfur atoms in aromatic rings (*S_aSa_*) have a negative influence on the DF value. It should be noted that, in the latter case, some of the training and test inactive compounds possess it, while all active compounds lack this functional group in their structure (the structure of all compounds as well as bibliographic references about their activity can be found in Tables S1 and S2).

DF_MRSA2_ involves one valence charge index (^3^*J^V^*) and three electrotopological indices (*S_=C<_*, *S_>CH-_* and *S_Cl_*). In this case, the presence of sp^2^ carbons (*S_=C<_*) and chlorine atoms (*S_Cl_*) increases its value, while the presence of sp^3^ carbons (*S_>CH-_*), and sp^2^ oxygens (*S_=O_*) decreases it. Regarding the charge index, the value of the DF is influenced negatively by the topological charge present in third-order sub-pseudographs (^3^*J^V^*). This type of index describes the distribution of the global charge in the molecule through the evaluation of charge transfer between pairs of atoms. Moreover, since it is also a valence index, it also considers heteroatoms and multiple bonds.

DF_MRSA3_ involves two electrotopological indices (*S_>N-_* and *S_Cl_*), one charge index (^3^*J*) and one geometric index (*PR2*). In this case, the value of the equation increases due to the presence of chlorine atoms (*S_Cl_*) and the geometric index *PR2*. Geometric indices are related to the shape and molecular surface [38]. The *PR2* index in particular counts the number of pairs of branches (understanding as branches the points that contain three or more vertices) separated by two axes. This means that the presence of ramifications favors the anti-MRSA activity. On the other hand, the value of the equation is negatively influenced by the presence of tertiary amine groups (*S_>N-_*) and very negatively by the topological charge in third-order sub-graphs (^3^*J*).

DF_MRSA4_ involves one connectivity index (^3^*χ_ch_*), two electrotopological indices (*S_Cl_* and *S_=CH-_*), and one charge index (^3^*J*). The equation also shows a clear dependence of the activity relative to the Kier–Hall chain-type third-order index (^3^*χ_ch_*), which implies that the presence of a cyclopropyl group greatly enhances the activity against MRSA. Most of the active compounds have this group. On the contrary, the inactive compounds generally lack this group (see Tables S1 and S2). This group has been proven to have a positive influence on the activity of a large number and variety of drugs [39]. The other index that has a positive influence on the value of the equation is *S_Cl_*, meaning that the presence of chlorine atoms favors the anti-MRSA activity, while the presence of sp^2^ carbons (*S_=CH-_*) is detrimental for such activity. The topological charge in third-order sub-graphs (^3^*J*) has also a negative influence on the anti-MRSA activity.

After analyzing all the equations, we found that the electrotopological index *S_Cl_* was common to all of them, with a positive influence in all cases. This indicates that the presence of this atom favors the anti-MRSA activity, as we concluded in one of our previous studies [34]. Foroumadi et al. [40] experimentally demonstrated the importance of this atom by substituting fluorine or hydrogen for chlorine in positions 6 and/or 8 from the double aromatic ring within the 4-quinolone structure. This change drastically increased the in vitro activity against two clinically isolated strains of MRSA and other bacterial species in numerous analog compounds.

**Figure 2:**
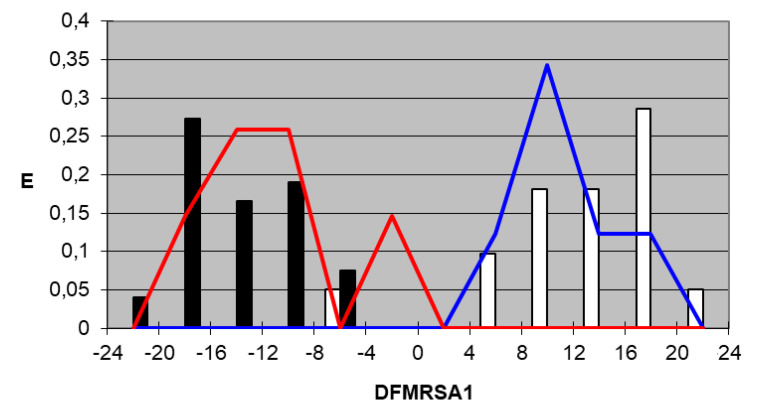
DF_MRSA1_ pharmacological distribution diagram. Black bars: Training Inactives. White bars: Training Actives. Red curves: Test Inactives. Blue curves: Test Actives.

**Figure 3:**
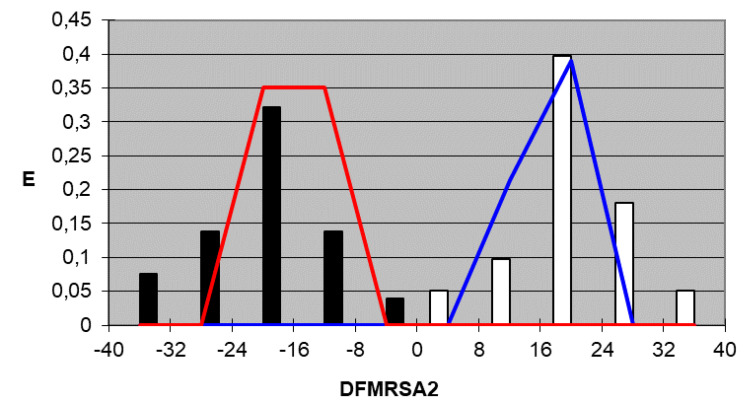
DF_MRSA2_ pharmacological distribution diagram. Black bars: Training Inactives. White bars: Training Actives. Red curves: Test Inactives. Blue curves: Test Actives.

**Figure 4:**
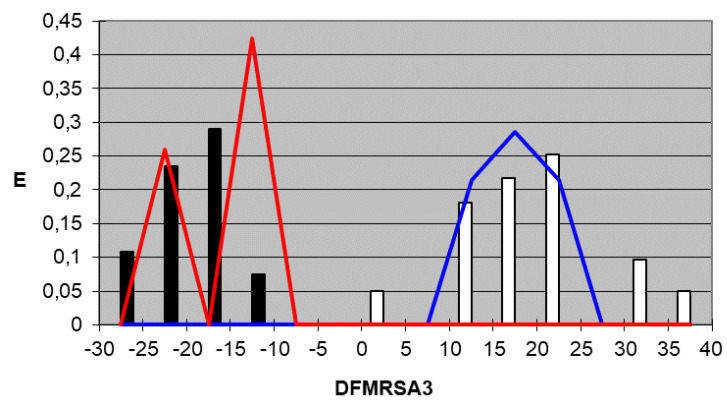
DF_MRSA3_ pharmacological distribution diagram. Black bars: Training Inactives. White bars: Training Actives. Red curves: Test Inactives. Blue curves: Test Actives.

**Figure 5:**
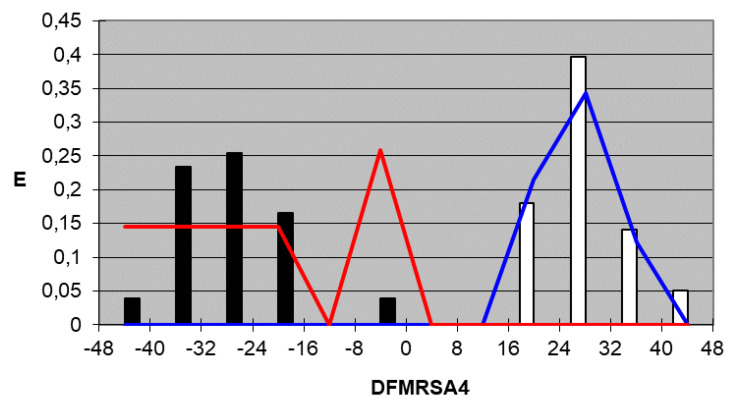
DF_MRSA4_ pharmacological distribution diagram. Black bars: Training Inactives. White bars: Training Actives. Red curves: Test Inactives. Blue curves: Test Actives.

We plotted the corresponding pharmacological distribution diagrams (PDD) for every function in order to visualize the values of the function in which the probability of classifying a compound as active or inactive is maximum—in other words, to find areas where the overlap between the two groups of compounds is minimal. The PDDs obtained for the DFs built along with the highest activity range for each function are shown in Figure 2, Figure 3, Figure 4 and Figure 5. Compounds with values below the range were considered inactive while compounds with values over the range were considered unclassified. Thus, the value ranges derived from these PDDs establish the applicability domain for each of them [41].

DF_MRSA1_*Highest Activity Expectancy* = 4–24

DF_MRSA2_*Highest Activity Expectancy* = 0–40

DF_MRSA3_*Highest Activity Expectancy* = 10–40

DF_MRSA4_*Highest Activity Expectancy* = 16–48

When PDDs are used, the accuracy for active compounds decreases, whereas that for inactive ones increases, so the probability of selecting a false active compound after applying the PDD filter decreases. The average percentage for the four equations of correctly classified inactive compounds is 100% for both the training and test group, and the average percentage of accurately classified active compounds is 97.4% for the training group and 100% for the test group. Table 5 and Table 6 summarize the classification of the results obtained for all functions selected for both training groups, the active group and inactive group, respectively, and Table 7 summarizes the results for the test groups. As can be inferred from the tables, the training and test groups exhibit an average overall accuracy of 99.1%.

## 4. Materials and Methods

### 4.1. Compound Selection

We collected in vitro activity information on quinolones and quinolone-like compounds using search engines such as ISI Web of Science, Medline, and SciFinder (Caplus). We only considered as valid those activity data from in vitro tests conducted according to the criteria of the CLSI [42].

We finally selected in vitro activity data of 56 quinolones and structurally related compounds (Figure 1) against MRSA, which we classified into two groups, active (26 compounds) and inactive (30 compounds), all of which had a 4-quinolone or a closely related structure (see Tables S1 and S2).

To consider a compound as active, it should have a minimum inhibitory concentration (MIC) equal to or under 1 mg/mL against MRSA, while those compounds considered inactive should have an MIC equal to or over 16 mg/mL against MRSA. Compounds with MICs between 1 and 16 mg/mL against MRSA were not included in the study. Regarding stereoisomers, if any of them were active, they were included in the active group. If all individual stereoisomers or the mixture of them in any ratio were inactive, they were included as a single graph in the inactive group.

### 4.2. Topological Descriptors

We calculated 301 topological indices for all 56 molecules. Those indices with values of 0 or with identical values for all compounds were removed. Finally, all compounds were characterized by a total of 136 non-redundant, significant descriptors specific to each molecule. These descriptors do not contain 3D parameters. The description of all the molecular descriptors can be found in Table S3 along with their definitions and references. Using MOLCONN-Z [43] and DESMOL13 [44] programs, we computed the adjacency topological matrix obtained from the hydrogen-depleted chemical pseudographs, previously drawn with the ChemBioDraw Ultra 12.0 molecule-editing program of the ChemBioOffice 2010 package.

### 4.3. Linear Discriminant Analysis (LDA)

LDA is a pattern recognition method that allows the classification of a compound in a given group or category (e.g., active and inactive) based on a combination of variables (e.g., topological indices). Based on the Fisher–Snedecor parameter F, which relates the variance explained by the equation to the residual variance, we chose the variables used to compute the linear classification functions in a stepwise manner. At each step, the variable with the greater value of F (i.e., the variable that causes the larger contribution to the differentiation of groups in the discriminant function) is entered. On the other hand, selected variables with a small value of F (i.e., variables that lower the statistical significance of the classification function) are removed.

The percentage of correct classifications attained for each set assesses the discriminant ability of each DF. The classification criterion is the minimal Mahalanobis distance (distance of each case to the mean of all the cases in a category). The quality of the discriminant function was evaluated through Wilk’s U-statistical parameter, λ, which was obtained by a multivariate analysis of variance that tests the equality of group means for the variable in the discriminant model.

The BMDP 7M Biomedical package [45] was the software used for the LDA study. The program randomly chooses compounds for the training and test groups. With the training group, a predictive mathematical model relating activity to structural descriptors is obtained. Internal validation is performed by the Jack-Knife (JK) method. Finally, the program performs external validation by applying the classification function to the test group and calculating the percentage of classification success.

### 4.4. Pharmacological Distribution Diagrams (PDD)

PDDs are histogram-like plots of connectivity functions used to determine the intervals of the discriminant function in which the expectancy, E, to find active compounds is maximum. In these plots, expectancies appear on the ordinate axis. For an arbitrary interval of values of a given function, we can define the expectancy of activity as Ea = a/(i + 1), where “a” is the number of active compounds in the interval divided by the total number of active compounds, and “i” is the number of inactive compounds. The expectancy of inactivity is defined in a symmetrical way, as Ei = i/(a + 1) [41]. This representation provides good visualization of the regions of minimum overlap and helps selecting intervals in the abscissa axis with maximum probability of finding active compounds.

PDDs allowed us to carry out the assignment of thresholds useful to discriminate active from inactive compounds with the highest probability of success.

## 5. Conclusions

Currently, the development of resistance of microorganisms such as *Staphylococcus aureus* is one of the most important problems that has appeared in recent years in the treatment of infectious diseases. Molecular topology has been demonstrated to be a useful methodology for identifying new compounds with antimicrobial activity against MRSA. By combining it with LDA, we developed four mathematical–topological equations with outstanding discriminant ability according to the success rates. These results are supported by internal and external validation performed on all functions, as well as by all the statistical parameters, which can be considered very satisfactory in all cases.

We can conclude that the equations obtained in this study confirm molecular topology as a powerful and efficient tool in the discrimination of anti-MRSA activity, offering new insights in the search for new compounds with this specific activity as opposed to classical structure–activity relationships.

## Figures and Tables

**Table 1:** Classification matrix for DF_MRSA1_.

Group	Active	Inactive	% Success
Training active	18	1	94.7
Training inactive	0	24	100
Test active	7	0	100
Test inactive	0	6	100
JK ^1^ Training active	18	1	94.7
JK ^1^ Training inactive	0	24	100

^1^ Jack-Knife method.

**Table 2:** Classification matrix for DF_MRSA2_.

Group	Active	Inactive	% Success
Training active	19	0	100
Training inactive	0	24	100
Test active	7	0	100
Test inactive	0	6	100
JK Training active	18	1	94.7
JK Training inactive	0	24	100

**Table 3:** Classification matrix for DF_MRSA3_.

Group	Active	Inactive	% Success
Training active	19	0	100
Training inactive	0	24	100
Test active	7	0	100
Test inactive	0	6	100
JK Training active	19	0	100
JK Training inactive	0	24	100

**Table 4:** Classification matrix for DF_MRSA4_.

Group	Active	Inactive	% Success
Training active	19	0	100
Training inactive	0	24	100
Test active	7	0	100
Test inactive	0	6	100
JK Training active	19	0	100
JK Training inactive	0	24	100

**Table 5:** Results obtained after combining LDA and PDD for the DFs. Training group: actives.

Compound	DF_MRSA1_^1^	Clas ^2^	DF_MRSA2_^1^	Clas ^2^	DF_MRSA3_^1^	Clas ^2^	DF_MRSA4_^1^	Clas ^2^
11act4	16.626	+	16.357	+	19.694	+	27.203	+
11act5	11.683	+	19.403	+	20.222	+	31.400	+
14act5	15.581	+	21.126	+	20.144	+	27.456	+
17act4	16.644	+	23.153	+	19.97	+	27.456	+
18act4	16.891	+	25.075	+	20.329	+	31.400	+
21act5	5.8776	+	16.021	+	18.056	+	18.668	+
22act4	11.789	+	20.928	+	13.000	+	20.577	+
28act4	17.471	+	26.089	+	20.238	+	31.400	+
30act4	15.632	+	22.201	+	16.962	+	24.358	+
34act5	17.661	+	24.684	+	32.461	+	39.959	+
35act5	17.614	+	23.094	+	32.469	+	39.959	+
4act5	17.676	+	23.776	+	23.800	+	34.920	+
5j-act1	21.524	+	36.244	+	36.171	+	42.102	+
5n-act1	11.424	+	18.699	+	11.661	+	25.305	+
5o-act1	9.171	+	8.2196	+	16.414	+	25.681	+
5q-act1	13.671	+	15.283	+	14.049	+	23.702	+
5r-act1	13.698	+	16.238	+	12.881	+	26.177	+
DX-619	−5.262	-	4.795	+	0.783	-	18.497	+
Sitafloxacin	6.404	+	26.299	+	24.666	+	31.369	+

^1^ Value of the DF for each compound. ^2^ The compounds are classified as active (+) if the DF value is within its highest expectancy range or inactive (-) for values outside of this range.

**Table 6:** Results obtained after combining LDA and PDD for the DFs. Training group: inactives.

Compound	DF_MRSA1_	Clas	DF_MRSA2_	Clas	DF_MRSA3_	Clas	DF_MRSA4_	Clas
5i-in1	−10.922	-	−21.009	-	−17.353	-	−31.118	-
in1-27j	−22.140	-	−32.034	-	−19.221	-	−19.988	-
in10-4Ib	−16.168	-	−22.602	-	−15.965	-	−26.738	-
in2-4b	−14.542	-	−18.473	-	−16.944	-	−23.370	-
in1-21a	−16.970	-	−14.755	-	−15.335	-	−35.360	-
in104IIa	−12.625	-	−17.332	-	−20.429	-	−34.317	-
in104IIb	−19.293	-	−21.486	-	−17.198	-	−34.565	-
in2-4e	−11.261	-	−19.599	-	−17.474	-	−30.255	-
in2-4f	−17.167	-	−18.987	-	−23.238	-	−28.553	-
in4-2a	−10.009	-	−18.479	-	−21.815	-	−18.213	-
in5-5d	−9.646	-	−15.358	-	−10.418	-	−4.134	-
in5-5e	−16.077	-	−18.117	-	−19.044	-	−32.963	-
in5-5f	−16.923	-	−27.682	-	−18.119	-	−31.946	-
in5-5i	−17.294	-	−26.764	-	−21.206	-	−31.331	-
in5-5k	−16.676	-	−16.353	-	−23.087	-	−29.665	-
in5-5l	−17.522	-	−25.918	-	−22.162	-	−28.649	-
in6-11a	−8.490	-	−13.852	-	−16.405	-	−28.032	-
in6-11c	−8.617	-	−21.238	-	−22.280	-	−37.466	-
in6-7c	−15.449	-	−32.295	-	−14.505	-	−19.063	-
in6-7e	−5.119	-	−6.883	-	−16.149	-	−19.063	-
in7pge61	−4.877	-	−13.953	-	−28.176	-	−34.778	-
in8-4b	−14.251	-	−20.785	-	−25.718	-	−36.492	-
in8-4c	−14.397	-	−19.425	-	−24.568	-	−35.308	-
in9-4	−16.367	-	−26.331	-	−25.571	-	−40.321	-

**Table 7:** Results obtained after combining LDA and PDD for DF1–4. Test group.

**Actives**								
**Compound**	**DF_MRSA1_**	**Clas**	**DF_MRSA2_**	**Clas**	**DF_MRSA3_**	**Clas**	**DF_MRSA4_**	**Clas**
10act5	11.841	+	16.946	+	18.803	+	26.469	+
13act4	17.105	+	21.453	+	21.328	+	32.447	+
15act4	15.936	+	22.277	+	20.086	+	27.456	+
19act5	11.843	+	20.242	+	19.797	+	27.456	+
28act5	6.900	+	8.470	+	13.988	+	17.946	+
5m-act1	11.522	+	17.914	+	13.113	+	23.108	+
5p-act1	9.293	+	9.373	+	16.326	+	29.218	+
**Inactives**								
**Compound**	**DF_MRSA1_**	**Clas**	**DF_MRSA2_**	**Clas**	**DF_MRSA3_**	**Clas**	**DF_MRSA4_**	**Clas**
25aArt13	−13.76	-	−22.394	-	−14.901	-	−47.111	-
in8-4a	−14.613	-	−19.761	-	−22.232	-	−29.320	-
in5-5g	−10.006	-	−14.539	-	−13.525	-	−3.536	-
in5-5h	−16.448	-	−17.199	-	−22.131	-	−32.348	-
in5-5j	−10.225	-	−13.780	-	−14.556	-	−0.929	-
in7pge52	−2.362	-	−12.941	-	−12.566	-	−20.154	-

## Data Availability

The date presented in this study are available in the Supplementary material.

## Supplementary Materials

The following are available online at https://www.mdpi.com/article/10.3390/ijms22115823/s1, Table S1: Active group used in the discriminant functions: Paper name/code, IUPAC name & structure and bibliographic references about activity for each compound, Table S2: Inactive group used in the discriminant functions: Paper name/code, IUPAC name & structure and bibliographic references about activity for each compound, Table S3: Symbols and Definitions of Topological Indices used with DESMOL13 and MOLCONN-Z programs.
