# Supplementary material for: Molecular Topology for the Search of New Anti-MRSA Compounds
Source: Int J Mol Sci. 2021 May 29;22(11):5823. doi: 10.3390/ijms22115823 (PMC8199290; doi:10.3390/ijms22115823)
Supplement: Supplementary file 1 [file ijms-22-05823-s001.zip › Table S1.pdf]

Table S1. Active group used in the discriminant functions: Paper name/code, IUPAC name, structure and bibliographic references about activity for each compound.

Paper name/code	IUPAC name	Structure	References
11act4	3-(9-cyclopropyl-6-fluoro-3,4-dioxo-2,3,4,9-tetrahydroisothiazolo[5,4-b]quinolin-7-yl)benzonitrile	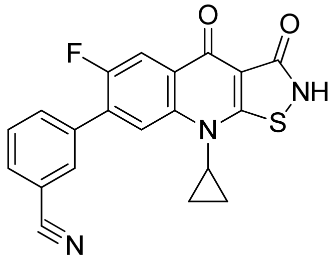	Wiles JA, Wang Q, Lucien E, Hashimoto A, Song Y, Cheng J, Marlor CW, Ou Y, Podos SD, Thanassi JA, Thoma CL, Deshpande M,Pucci MJ, Bradbury BJ. Isothiazoloquinolones containing functionalized aromatic hydrocarbons at the 7-position: synthesis and in vitro activity of a series of potent antibacterial agents with diminished cytotoxicity in human cells. *Bioorg. Med. Chem. Lett.* **2006**; *16(5)*: 1272-6.
13act4	2-(4-(9-cyclopropyl-6-fluoro-3,4-dioxo-2,3,4,9-tetrahydroisothiazolo[5,4-b]quinolin-7-yl)phenyl)acetonitrile	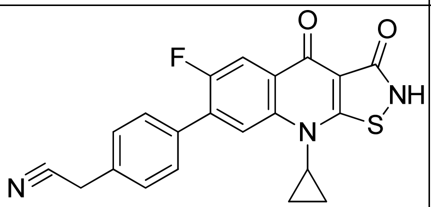	Idem
15act4	9-cyclopropyl-6-fluoro-7-(3-fluorophenyl)isothiazolo[5,4-*b*]quinoline-3,4(2*H*,9*H*)-dione	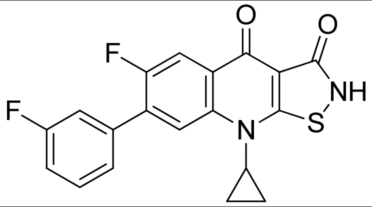	Idem
17act4	9-cyclopropyl-6-fluoro-7-(3-hydroxyphenyl)isothiazolo[5,4-b]quinoline-3,4(2H,9H)-dione	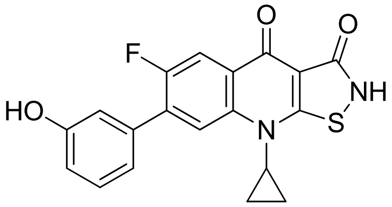	Idem
18act4	9-cyclopropyl-6-fluoro-7-(4-hydroxyphenyl)isothiazolo[5,4-b]quinoline-3,4(2H,9H)-dione	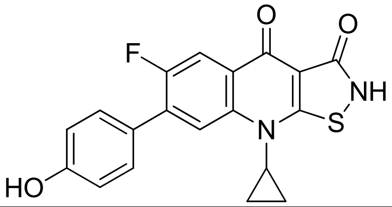	Idem
22act4	9-cyclopropyl-6-fluoro-7-(4-hydroxy-3-methoxyphenyl)isothiazolo[5,4-b]quinoline-3,4(2H,9H)-dione	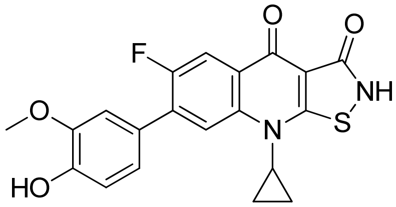	Idem

28act4	7-(4-aminophenyl)-9-cyclopropyl-6-fluoroisothiazolo[5,4-*b*]quinoline-3,4(2*H*,9*H*)-dione	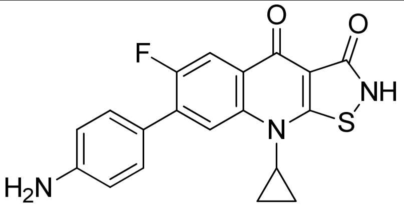	Idem
30act4	7-(3-amino-4-fluorophenyl)-9-cyclopropyl-6-fluoroisothiazolo[5,4-b]quinoline-3,4(2H,9H)-dione	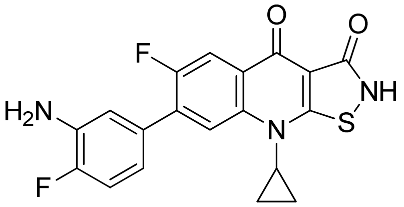	Idem
4act5	9-cyclopropyl-6-fluoro-7-(pyridin-4-yl)isothiazolo[5,4-b]quinoline-3,4(2H,9H)-dione	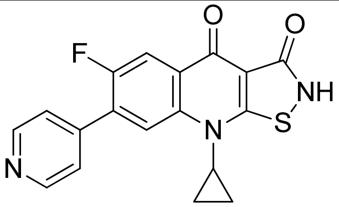	Wiles JA, Song Y, Wang Q, Lucien E, Hashimoto A, Cheng J, Marlor CW, Ou Y, Podos SD, Thanassi JA, Thoma CL, Deshpande M, Pucci MJ, Bradbury BJ. Biological evaluation of isothiazoloquinolones containing aromatic heterocycles at the 7-position: In vitro activity of a series of potent antibacterial agents that are effective against methicillin-resistant Staphylococcus aureus. *Bioorg. Med. Chem. Lett.* **2006**; *16(5)*: 1277-81.
10act5	9-cyclopropyl-6-fluoro-7-(2-methylpyridin-3-yl)isothiazolo[5,4-b]quinoline-3,4(2H,9H)-dione	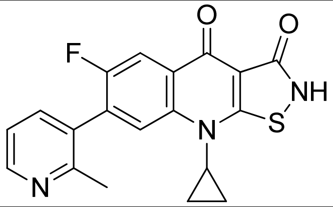	Idem
11act5	9-cyclopropyl-6-fluoro-7-(6-methylpyridin-3-yl)isothiazolo[5,4-b]quinoline-3,4(2H,9H)-dione	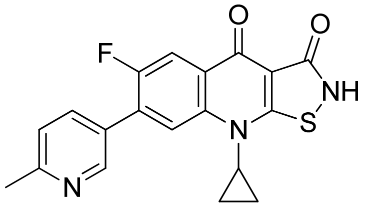	Idem
14act5	9-cyclopropyl-6-fluoro-7-(2-fluoropyridin-4-yl)isothiazolo[5,4-b]quinoline-3,4(2H,9H)-dione	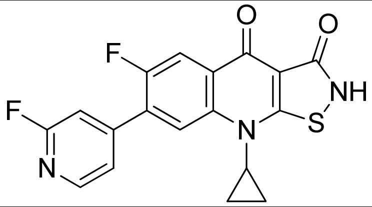	Idem
19act5	9-cyclopropyl-6-fluoro-7-(2-methylpyridin-4-yl)isothiazolo[5,4-*b*]quinoline-3,4(2*H*,9*H*)-dione	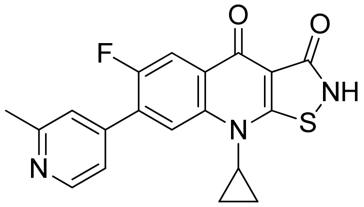	Idem

21act5	9-cyclopropyl-7-(2,6-dimethylpyridin-4-yl)-6-fluoroisothiazolo[5,4-*b*]quinoline-3,4(2*H*,9*H*)-dione	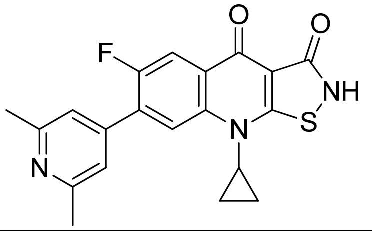	Idem
28act5	9-cyclopropyl-7-(2,4-dimethoxypyrimidin-5-yl)-6-fluoroisothiazolo[5,4-*b*]quinoline-3,4(2*H*,9*H*)-dione	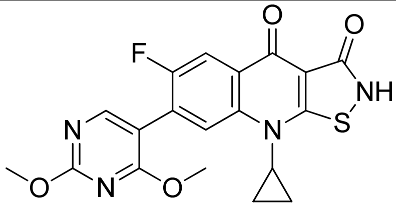	Idem
34act5	9-cyclopropyl-6-fluoro-7-(isoquinolin-6-yl)isothiazolo[5,4-b]quinoline-3,4(2H,9H)-dione	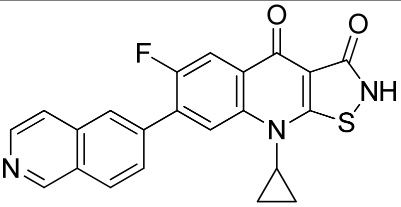	Idem
35act5	9-cyclopropyl-6-fluoro-7-(quinolin-6-yl)isothiazolo[5,4-b]quinoline-3,4(2H,9H)-dione	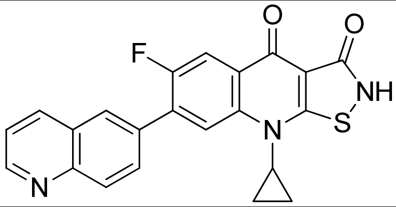	Idem
5j-act1	(E)-1-cyclopropyl-7-(4-(2-(2,4-dichlorophenyl)-2-(methoxyimino)ethyl)piperazin-1-yl)-6-fluoro-4-oxo-1,4-dihydroquinoline-3-carboxylic acid	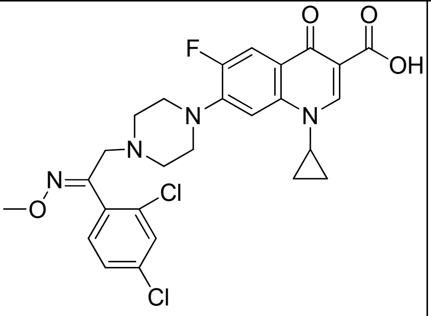	Foroumadi A, Ghodsi S, Emami S, Najjari S, Samadi N, Faramarzi MA, Beikmohammadi L, Shirazi FH, Shafiee A. Synthesis and antibacterial activity of new fluoroquinolones containing a substituted N-(phenethyl)piperazine moiety. *Bioorg. Med. Chem. Lett.* **2006**; *16(13)*: 3499-3503.
5m-act1	(E)-7-(4-(2-(2-chlorophenyl)-2-(methoxyimino)ethyl)piperazin-1-yl)-1-cyclopropyl-6-fluoro-4-oxo-1,4-dihydroquinoline-3-carboxylic acid	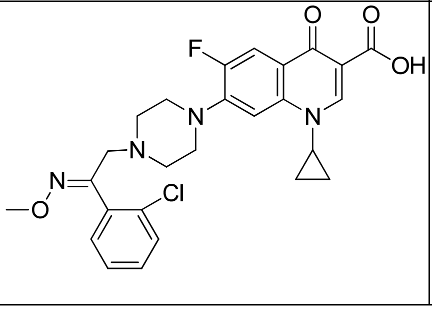	Idem

5n-act1	(E)-7-(4-(2-(4-chlorophenyl)-2-(methoxyimino)ethyl)piperazin-1-yl)-1-cyclopropyl-6-fluoro-4-oxo-1,4-dihydroquinoline-3-carboxylic acid	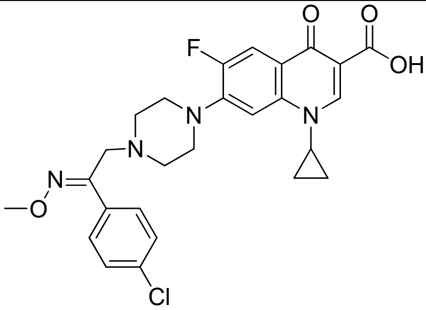	Idem
5o-act1	7-(4-(2-(2-chlorophenyl)-2-oxoethyl)piperazin-1-yl)-1-cyclopropyl-6-fluoro-4-oxo-1,4-dihydroquinoline-3-carboxylic acid	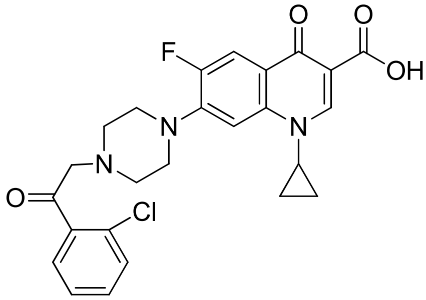	Idem
5p-act1	7-(4-(2-(4-chlorophenyl)-2-oxoethyl)piperazin-1-yl)-1-cyclopropyl-6-fluoro-4-oxo-1,4-dihydroquinoline-3-carboxylic acid	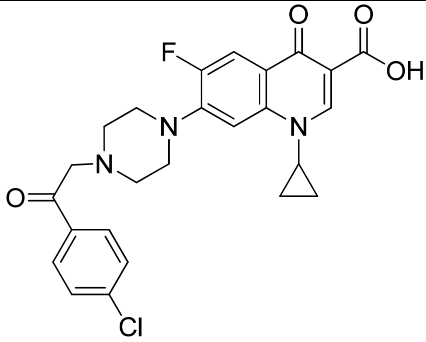	Idem
5q-act1	(E)-7-(4-(2-(2-chlorophenyl)-2-(hydroxyimino)ethyl)piperazin-1-yl)-1-cyclopropyl-6-fluoro-4-oxo-1,4-dihydroquinoline-3-carboxylic acid	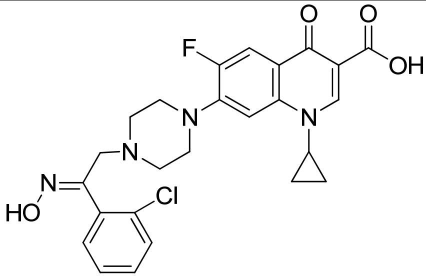	Idem
5r-act1	(E)-7-(4-(2-(4-chlorophenyl)-2-(hydroxyimino)ethyl)piperazin-1-yl)-1-cyclopropyl-6-fluoro-4-oxo-1,4-dihydroquinoline-3-carboxylic acid	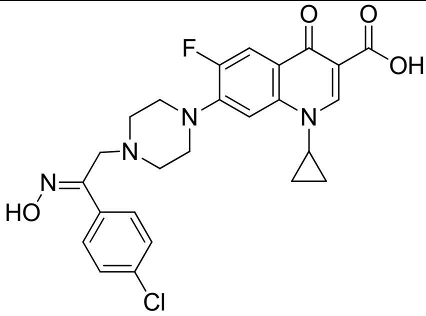	Idem

DX-619	7-((R)-3-(1-aminocyclopropyl)pyrrolidin-1-yl)-1-((1R,2S)-2-fluorocyclopropyl)-8-methoxy-4-oxo-1,4-dihydroquinoline-3-carboxylic acid	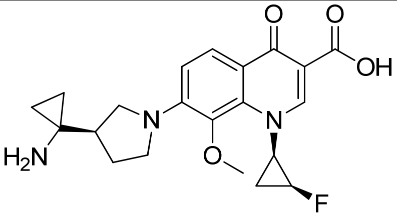	Fujikawa K, Chiba M, Tanaka M, Sato K. In vitro antibacterial activity of DX-619, a novel des-fluoro(6) quinolone. *Antimicrob. Agents Chemother.* **2005**; *49(7)*: 3040-5.
Sitafloxacin	7-((S)-7-amino-5-azaspiro[2.4]heptan-5-yl)-8-chloro-6-fluoro-1-((1S,2R)-2-fluorocyclopropyl)-4-oxo-1,4-dihydroquinoline-3-carboxylic acid	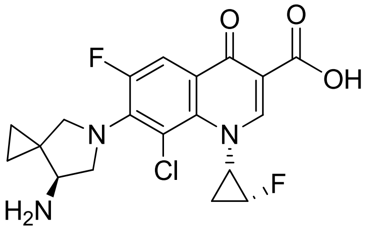	Milatovic D, Schmitz FJ, Brisse S, Verhoef J, Fluit AC. In Vitro Activities of Sitafloxacin (DU-6859a) and Six Other Fluoroquinolones against 8,796 Clinical Bacterial Isolates. *Antimicrob. Agents Chemother.* **2000**; *44(4)*: 1102-7.
