# Supplementary material for: Molecular Topology for the Search of New Anti-MRSA Compounds
Source: Int J Mol Sci. 2021 May 29;22(11):5823. doi: 10.3390/ijms22115823 (PMC8199290; doi:10.3390/ijms22115823)
Supplement: Supplementary file 1 [file ijms-22-05823-s001.zip › Table S2.pdf]

Table S2. Inactive group used in the discriminant functions: Paper name/code, IUPAC name, structure and bibliographic references about activity for each compound.

Paper name/code	IUPAC name	Structure	References
5i-in1	(E)-7-(4-(2-(2,4-difluorophenyl)-2-(methoxyimino)ethyl)piperazin-1-yl)-1-ethyl-6-fluoro-4-oxo-1,4-dihydro-1,8-naphthyridine-3-carboxylic acid	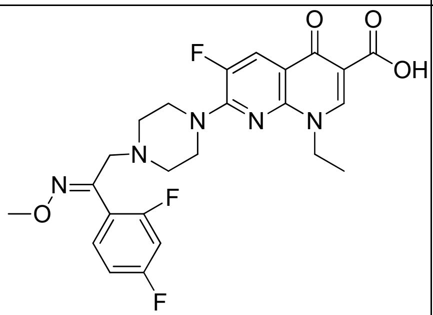	Foroumadi A, Ghodsi S, Emami S, Najjari S, Samadi N, Faramarzi MA, Beikmohammadi L, Shirazi FH, Shafiee A. Synthesis and antibacterial activity of new fluoroquinolones containing a substituted N-(phenethyl)piperazine moiety. *Bioorg. Med. Chem. Lett.* **2006**; *16(13)*: 3499-3503.
in6-7c	5-ethyl-6-hydroxy-8-oxo-5,8-dihydro-[1,3]dioxolo[4,5-g]quinoline-7-carboxylic acid	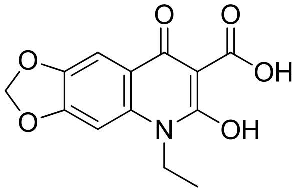	Mitscher LA, Gracey HE, Clark GW 3rd, Suzuki T. Quinolone antimicrobial agents. 1. Versatile new synthesis of 1-alkyl-1,4-dihydro-4-oxo-3-quinolinecarboxylic acids. *J. Med. Chem.* **1978**; *21(5)*: 485-9.
in6-7e	5-ethyl-6-methyl-8-oxo-5,8-dihydro-[1,3]dioxolo[4,5-g]quinoline-7-carboxylic acid	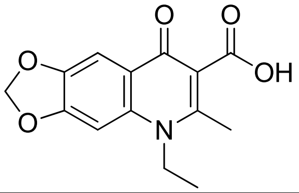	Idem
in6-11a	1-ethyl-6-methyl-4-oxo-1,2,3,4-tetrahydroquinoline-3-carboxylic acid	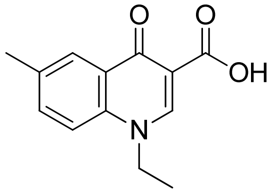	Idem
in6-11c	1-ethyl-8-methyl-4-oxo-1,2,3,4-tetrahydroquinoline-3-carboxylic acid	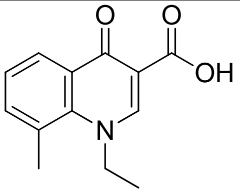	Idem
in2-4b	1-(2-(difluoro(hydroxy)methyl)phenyl)-6-fluoro-7-(4-methylpiperazin-1-yl)-4-oxo-1,4-dihydroquinoline-3-carboxylic acid	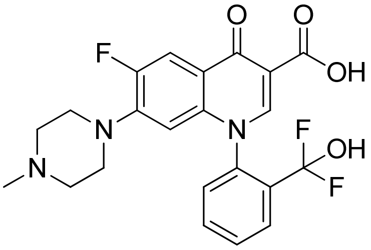	Xiao W, Krishnan R, Lin YI, Delos Santos EF, Kuck NA, Babine RE, Lang SA Jr. Synthesis and in vitro antibacterial activity of some 1-(difluoromethoxyphenyl)quinolone-3-carboxylic acids. *J. Pharm. Sci.* **1989**; *78(7)*; 585-8.

in2-4e	1-(3,4-bis(difluoro(hydroxy)methyl)phenyl)-6-fluoro-4-oxo-7-(piperazin-1-yl)-1,4-dihydroquinoline-3-carboxylic acid	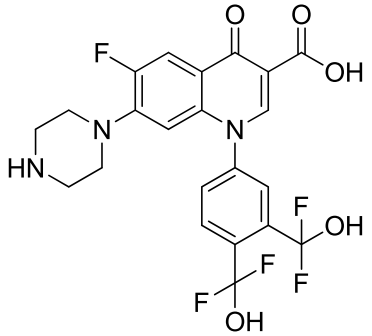	Idem
in2-4f	1-(3,4-bis(difluoro(hydroxy)methyl)phenyl)-6-fluoro-7-(4-methylpiperazin-1-yl)-4-oxo-1,4-dihydroquinoline-3-carboxylic acid	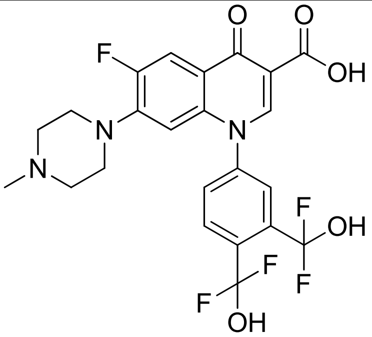	Idem
in10-4Ib	7-(2,3-dimethylthiazolidin-4-yl)-1-ethyl-4-oxo-1,4-dihydroquinoline-3-carboxylic acid	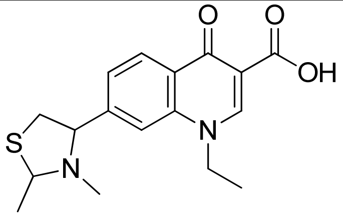	Zhang M, Haemers A, Vanden Berghe D, Pattyn S, Bollaert W, Levshin I. Quinolone antibacterials. 1. 7-(2-Substituted-4-thiazolyl- and -thiazolidinyl)quinolones. *J. Heterocyclic Chem.* **1991**; *22(38)*: 673-83.
in10-4IIa	1-ethyl-6-fluoro-7-(3-methylthiazolidin-4-yl)-4-oxo-1,4-dihydroquinoline-3-carboxylic acid	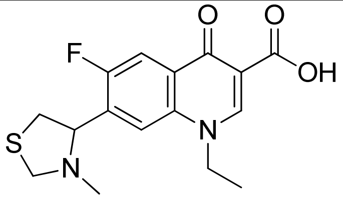	Idem
in10-4IIb	7-(2,3-dimethylthiazolidin-4-yl)-1-ethyl-6-fluoro-4-oxo-1,4-dihydroquinoline-3-carboxylic acid	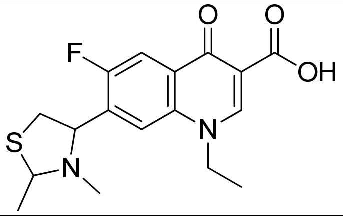	Idem
in9-4	7-ethyl-3-methyl-4-oxo-4,7-dihydroisothiazolo[5,4-b]pyridine-5-carboxylic acid	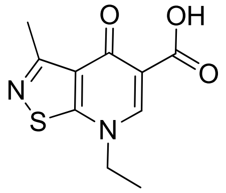	Richardson TO; Neale N, Carwell N. Synthesis and antibacterial activity of 7-ethyl-3-methyl-4,7-dihydro-4-oxoisothiazolo[5,4-b]pyridine-5-carboxylic acid. *J. Heterocyclic Chem.* **1995**; *32(1)*: 359-61

in1-21a	7-amino-4-(4-methoxyphenyl)-5-methyl-6-(4-methylpiperazin-1-yl)-1-oxo-1,4-dihydronaphthalene-2-carboxylic acid	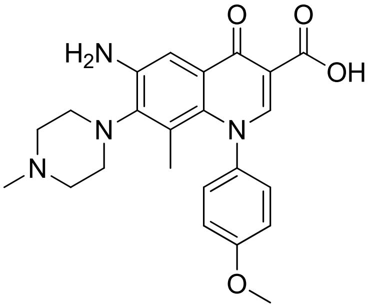	Cecchetti V, Fravolini A, Lorenzini MC, Tabarrini O, Terni P, Xin T. Studies on 6-aminoquinolones: synthesis and antibacterial evaluation of 6-amino-8-methylquinolones. *J. Med. Chem.* **1996**; *39(2)*: 436-45.
in1-27j	1-cyclopropyl-6-(dimethylamino)-8-methyl-7-(4-methylpiperidin-1-yl)-4-oxo-1,4-dihydroquinoline-3-carboxylic acid	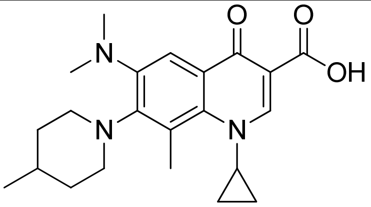	Idem
in4-2a	7-amino-4-cyclopropyl-5-ethyl-6-(4-methylpiperazin-1-yl)-1-oxo-1,4-dihydronaphthalene-2-carboxylic acid	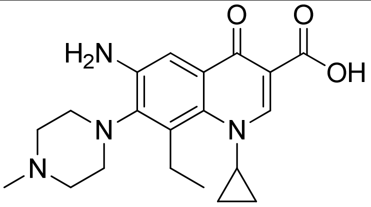	Cecchetti V, Tabarrini O, Sabatini S, Miao H, Filipponi E, Fravolini A. Studies on 6-aminoquinolones: synthesis and antibacterial evaluation of 6-amino-8-ethyl- and 6-amino-8-methoxyquinolones. *Bioorg. Med. Chem.* **1999**; *7(11)*: 2465-71.
in5-5d	1-cyclopropyl-6-fluoro-7-(4-(5-(2-nitrophenyl)-1,3,4-thiadiazol-2-yl)piperazin-1-yl)-4-oxo-1,2,3,4-tetrahydroquinoline-3-carboxylic acid	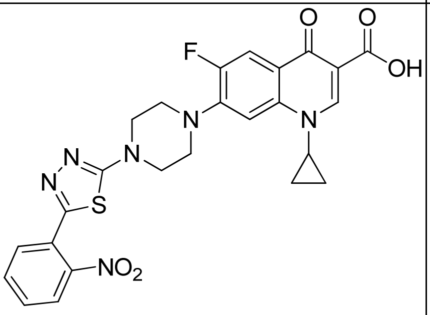	Foroumadi A, Soltani F, Moshafi MH, Ashraf-Askari R. Synthesis and in vitro antibacterial activity of some N-(5-aryl-1,3,4-thiadiazole-2-yl)piperazinyl quinolone derivatives. *Il Farmaco* **2003**; *58 (10)*: 1023-8.
in5-5e	1-ethyl-6-fluoro-7-(4-(5-(2-nitrophenyl)-1,3,4-thiadiazol-2-yl)piperazin-1-yl)-4-oxo-1,2,3,4-tetrahydroquinoline-3-carboxylic acid	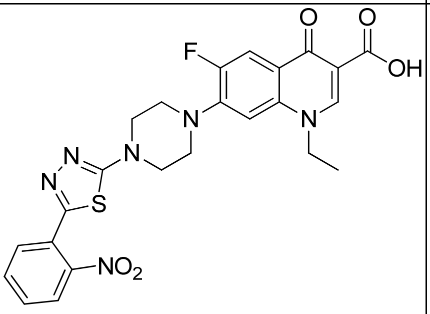	Idem
in5-5f	1-ethyl-6-fluoro-7-(4-(5-(2-nitrophenyl)-1,3,4-thiadiazol-2-yl)piperazin-1-yl)-4-oxo-1,2,3,4-tetrahydro-1,8-naphthyridine-3-carboxylic acid	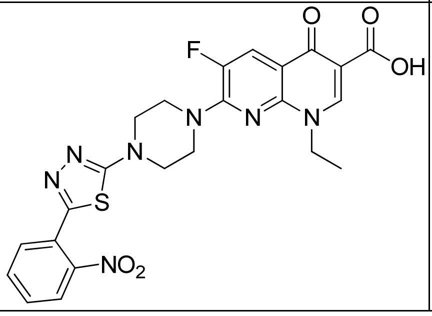	Idem

in5-5g	1-cyclopropyl-6-fluoro-7-(4-(5-(3-nitrophenyl)-1,3,4-thiadiazol-2-yl)piperazin-1-yl)-4-oxo-1,2,3,4-tetrahydroquinoline-3-carboxylic acid	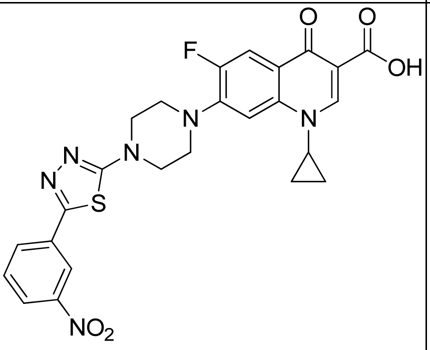	Idem
in5-5h	1-ethyl-6-fluoro-7-(4-(5-(3-nitrophenyl)-1,3,4-thiadiazol-2-yl)piperazin-1-yl)-4-oxo-1,2,3,4-tetrahydroquinoline-3-carboxylic acid	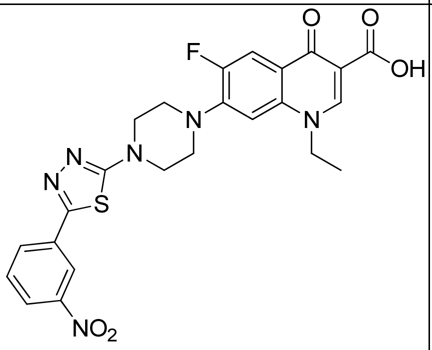	Idem
in5-5i	1-ethyl-6-fluoro-7-(4-(5-(3-nitrophenyl)-1,3,4-thiadiazol-2-yl)piperazin-1-yl)-4-oxo-1,2,3,4-tetrahydro-1,8-naphthyridine-3-carboxylic acid	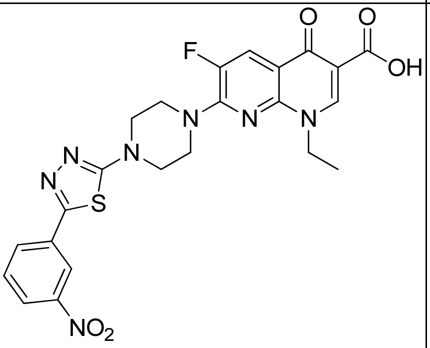	Idem
in5-5j	1-cyclopropyl-6-fluoro-7-(4-(5-(4-nitrophenyl)-1,3,4-thiadiazol-2-yl)piperazin-1-yl)-4-oxo-1,2,3,4-tetrahydroquinoline-3-carboxylic acid	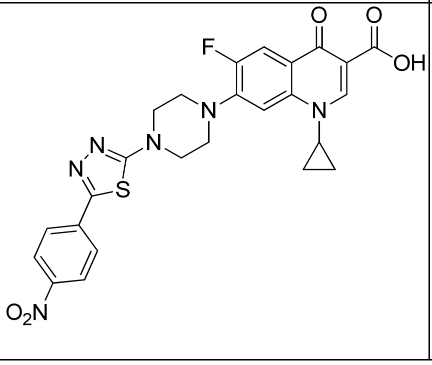	Idem

in5-5k	1-ethyl-6-fluoro-7-(4-(5-(4-nitrophenyl)-1,3,4-thiadiazol-2-yl)piperazin-1-yl)-4-oxo-1,2,3,4-tetrahydroquinoline-3-carboxylic acid	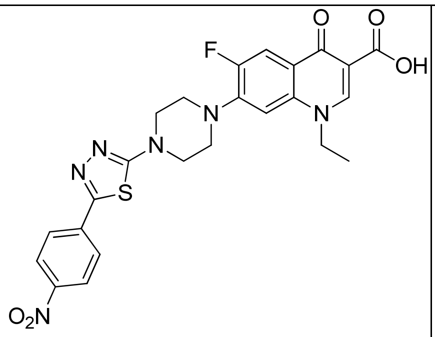	Idem
in5-5l	1-ethyl-6-fluoro-7-(4-(5-(4-nitrophenyl)-1,3,4-thiadiazol-2-yl)piperazin-1-yl)-4-oxo-1,2,3,4-tetrahydro-1,8-naphthyridine-3-carboxylic acid	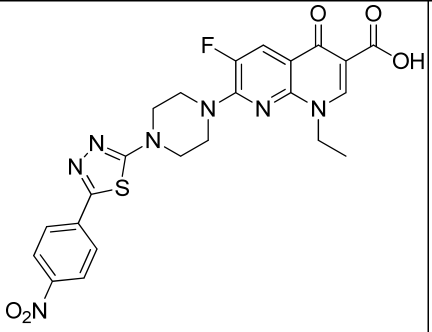	Idem
25aArt13	9-fluoro-3-methyl-10-(3-methylpiperazin-1-yl)-7-oxo-3,7-dihydro-2H-[1,4]oxazino[2,3,4-ij]quinoline-6-carboxylic acid	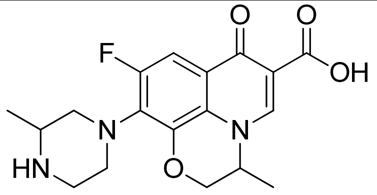	Liu B, Yang CH, Xu GY, Zhu YH, Cui JR, Wu XH, Xie YY. Syntheses of quinolone hydrochloride enantiomers from synthons (R)- and (S)-2-methylpiperazine. *Bioorg. Med. Chem.* **2005**; *13(7)*: 2451-8.
in8-4a	7-(4-(5-(benzylsulfonyl)-1,3,4-thiadiazol-2-yl)piperazin-1-yl)-1-ethyl-6-fluoro-4-oxo-1,4-dihydroquinoline-3-carboxylic acid	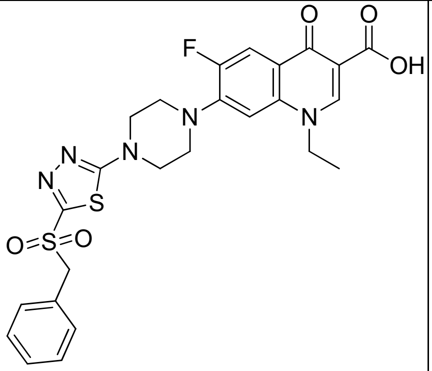	Foroumadi A, Emami S, Hassanzadeh A, Rajaee M, Sokhanvar K, Moshafi MH, Shafiee A. Synthesis and antibacterial activity of N-(5-benzylthio-1,3,4-thiadiazol-2-yl) and N-(5-benzylsulfonyl-1,3,4-thiadiazol-2-yl)piperazinyl quinolone derivatives. *Bioorg. Med. Chem. Lett.* **2005**; *15(20)*: 4488-92.
in8-4b	1-ethyl-6-fluoro-7-(4-(5-((2-nitrobenzyl)sulfonyl)-1,3,4-thiadiazol-2-yl)piperazin-1-yl)-4-oxo-1,4-dihydroquinoline-3-carboxylic acid	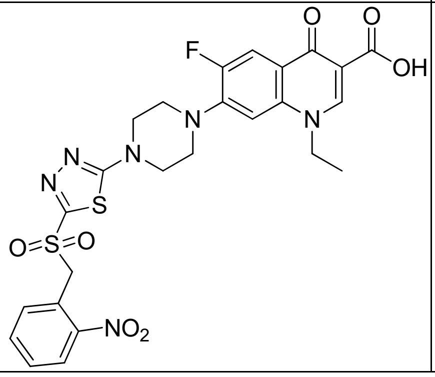	Idem

in8-4c	1-ethyl-6-fluoro-7-(4-(5-((3-nitrobenzyl)sulfonyl)-1,3,4-thiadiazol-2-yl)piperazin-1-yl)-4-oxo-1,4-dihydroquinoline-3-carboxylic acid	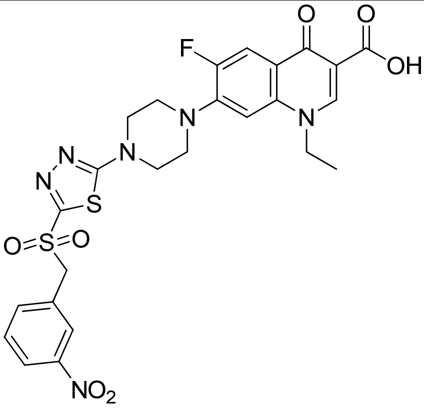	Idem
in7pge52	1-methyl-4-oxo-1,4-dihydroquinoline-3-carboxylic acid	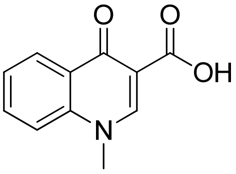	Macinga DR, Renick PJ, Makin KM, Ellis DH, Kreiner AA, Li M, Rupnik KJ, Kincaid EM, Wallace CD, Ledoussal B, Morris TW. Unique Biological Properties and Molecular Mechanism of 5,6-Bridged Quinolones. *Antimicrob. Agents Chemother.* **2003**; *47(8)*: 2526-37.
in7pge61	7,8-dimethyl-10-oxo-7,10-dihydro-1H-[1,3]dioxino[5,4-f]quinoline-9-carboxylic acid	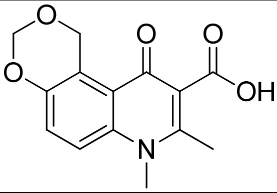	Idem
